# Job Type, Religion, and Muslim Gender as Predictors of Discrimination in Employment Settings

**DOI:** 10.1177/00332941221092666

**Published:** 2022-10-12

**Authors:** Kazhal Mansouri, Richard Perlow

**Affiliations:** 120459 University of Lethbridge Dhillon School of Business, Lethbridge, Alberta, Canada; 120459 University of Lethbridge Dhillon School of Business, Lethbridge, Alberta, Canada

**Keywords:** Employment discrimination, religious discrimination, selection, Muslim discrimination

## Abstract

Most employment discrimination research has focused on race and gender. The relatively fewer papers dealing with religion suggests that discrimination exists. We extend the literature by examining the effects of job type (public safety/non-public safety), religion (Muslim/non-Muslim) and Muslim gender on selection decisions. Participants ranked applicants and made judgments on trust and whether to interview applicants after evaluating seven resumes for either a shipping clerk or a security guard position. Participants rated Muslim applicants lower than non-Muslim applicants for the security guard position. We found no evidence of discrimination in the shipping clerk position. Perceived trust may be a possible explanation for some of the decisions people made. We also found that the Muslim female candidate was rated higher than the Muslim male candidate for the security guard position; no gender differences existed for the shipping clerk position. Our findings are consistent with the gender discrimination literature in that job type affected the extent to which religious-based discrimination occurred and the intersectionality literature/models specifying that combinations of demographics can impact judgments. One implication is the need to incorporate religion in discrimination interventions.

Employment discrimination occurs when employers differentiate among people based on job irrelevant factors such as race, sexual orientation, religion, gender, and age (Arvey, 1979; Chung, 2001).^1^ Typically, people discriminate unfairly based on prejudice and the stereotypes they hold (Arvey, 1986; Campion & Arvey, 1989). Studying employment discrimination is important because it reduces organizational commitment (Foley et al., 2005; Triana et al., 2010), negatively affects health and organizational citizenship (Triana et al., 2015), lowers job satisfaction and engagement (Foley et al., 2005; Kim, 2015), can lead to increased reports from women of work conflict and lower perceptions of power and job prestige (Gutek et al., 1996), and is prohibited in some jurisdictions by law (Canadian Human Rights Commission, 2000; Civil Rights Act of, 1964).

Most of the discrimination literature has focused on African Americans (e.g., Brown & Ford, 1977; Hughes & Dodge, 1997; Nord & Ting, 1994; Slaughter et al., 2002; Smith et al., 1978*) and women* (Heilman, 1980, 1983; Heilman & Martell, 1986; Heilman et al., 1988; Heilman & Saruwatari, 1979; Heilman & Stopeck, 1985). The race literature has *suggested that interviewers perceive* African-American applicants as less intelligent than whites (Frazer & Wiersma, 2001), that they have insufficient soft skills (Moss & Tilly, 1996), and have had less job security than white employees have (Durr & Logan, 1997). Heilman’s lack of fit model has been used to explain gender discrimination. The model specifies that evaluators assess the congruence between job requirements and the candidate’s knowledge, skills, and abilities. Evaluators emit biased judgments when their stereotypes of both people and job requirements influence their perceptions of congruence. The more stereotypes influence congruence perceptions, the greater the likelihood of discrimination occurring in selection, performance appraisal, compensation, and other HRM functions (see Cash et al., 1977). There is also evidence indicating that findings on gender discrimination as a function of job type generalizes to race (Stewart & Perlow, 2001). Although the literature cited above has increased our understanding of employment discrimination, gaps in our knowledge remain. Compared to race and gender, relatively little research has focused on religious discrimination. In our study, we extend the literature by examining Muslim discrimination as a function of job type.

Understanding religious discrimination in general, and Muslim discrimination in particular is important for several reasons. Allegations of discrimination can result in expensive lawsuits. Knowledge that a company may discriminate unfairly has adverse effects on the corporate brand. Developing a reputation as a discriminatory organization may impact the number of candidates from the targeted group applying for jobs in the organization that, in turn, might reduce the number of highly qualified people selected from the that group. In societal terms, discrimination results in underutilization of capable people in the workforce (Esses et al., 2014). The topic is also important given the increased number of religious discrimination complaints in some jurisdictions (e.g., from 1709 in FY 1997–2404 in FY 2020 - EEOC, 2021). The increasing number of complaints have led some to call for more research understanding the nature and causes of religious discrimination (Ghumman & Jackson, 2010; Ghumman et al., 2013; Ruggs, et al., 2013).

Studying Muslim discrimination is important for several reasons. Islam has the second largest number of adherents and it is one of the world’s fastest growing religions (Pew Research Center, 2015; 2017). For example, in Canada, the latest available data show that the number of Islam adherents increased nearly four-fold from approximately 253,300 in 1991 to 1,053,945 in 2011 and represents approximately 3.2% of the total Canadian population (Statistics Canada, 2011). Survey results reveal that people hold negative views of Muslims (YouGov, 2015). The Muslim population growth in locales such as Europe and the West coupled with the negative views people have of Muslims triggers the need to investigate the potential discrimination that may occur against them in employment settings.

## Muslim Discrimination

Survey data point to the existence of Muslim discrimination (Padela et al., 2016). For example, Statistics Canada (2021) reports there were 608 police-reported religious-related hate crimes in 2019. Of these, 296 (49%) targeted Jews; 181 (30%) targeted Muslims. There was a 9% increase in Muslim hate crimes in 2019 compared to 2018 (Statistics Canada, 2021). Research also documents the presence of employment-related discrimination in terms of call back rates (Adida et al., 2010; Valfort, 2020), job status (Ghumman & Jackson, 2008), job suitability (Doris et al., 2009) the presence or demonstration of religious affiliation (Padela et al., 2016; Valfort, 2020), the presence of negative candidate information (Park et al., 2009), attire (Unkelbach et al., 2010), attire in less diverse organizations (Ghumman & Ryan, 2013), and the absence of stereotype-inconsistent information (King & Ahmad, 2010). Research also shows that bias towards Muslims is not limited to North American evaluators (Di Stasio et al., 2021; Grigoryan, 2020; Strabac & Listhaug, 2008) and that bias exists in some media (Luqiu & Yang, 2018).

While the research cited above has contributed to understanding how people respond to Muslims, less is known about job context effects. Assuming there are individuals who view Muslims with suspicion and as people who cannot be trusted, perhaps Muslim applicants to public safety jobs or jobs that serve the public good might elicit discriminatory behavior. Unfortunately, we could not find much research examining this issue. We address this gap by examining religion and gender effects on two jobs that are differentially related to public safety.

## Theoretical Rationale

Several perspectives informed our hypotheses. The stereotype content model (Fiske, 2015, 2017; Lee & Fiske, 2006) is a framework useful in understanding how stereotyping might affect how Muslims compare to non-Muslims in some selection contexts. The model specifies that individuals form stereotypes of minorities, outgroups, and immigrants based on two dimensions: warmth and competence (Eagly & Kite, 1987; Fiske, 2015, 2017; Kil et al., 2019; Lee & Fiske, 2006; Strinić et al., 2020). Warmth, and its equivalent concept communion, is a dimension pertaining to the concern with the well-being of others (Eagly et al., 2020). Characteristics of people high in warmth/communion include trustworthiness, compassion, friendliness, and being well-intentioned (Agerström et al., 2012; Eagly et al., 2020; Fiske, 2015; Lee & Fiske, 2006). The stereotype classification within which evaluators place the warmth of minorities can impact evaluator decision-making.

Social identity theory (Ashforth & Mael, 1989; Tajfel & Turner, 1986) is another perspective explaining how discrimination occurs. People exhibit a tendency to classify themselves and others into social categories such as religion and gender. They do this, in part, to organize, manage, and understand their social environment including how they themselves and how others might behave (Hogg, 2000). One consequence of the classification process is the cognitive formation of groups to which the person belongs and groups to which the person doesn’t belong. Favoritism towards members of the person’s in-group and negative stereotyping of members belonging to the out-group can occur when the evaluator has a high level of emotional involvement with a particular social categorization (Brewer, 1995). Similarly, interpersonal attraction perspectives specify that people are attracted to others based on the similarity of factors such as attitudes, behaviors, and beliefs (Byrne, 1971; Hogg & Abrams, 1988; Kanter, 1977). Given the stereotypes and research results that describe Muslims as lacking warmth (Durante et al., 2017; Fiske, 2017), or possessing less warmth than other groups (Agerström et al., 2012), we would expect the evaluation of non-Muslim applicants to be more favorable than the evaluation Muslims applicants receive.

Operating under the assumption that certain jobs have features that are salient to employers (e.g., ensuring public safety) and given that choosing people for jobs is a decision-making activity, the decision-making literature is also of theoretical import because that literature specifies how saliency can lead to biased judgments. Kahneman and Tversky’s work (Kahneman & Tversky, 1972; Tversky & Kahneman, 1973) on heuristics is particularly useful in understanding discrimination. They claim that people take mental shortcuts when engaged in decision-making activities, and that the mental shortcuts people take can lead to systematic biased judgments. Specifically, people misjudge the frequency with which events occur based on how easily they can recall instances of those events. They also misjudge the probability of an event occurring based on the degree to which the event contains similar features with the parent population. Their work is particularly relevant during the early stages of the selection process because employers have limited information on job applicants during initial resume review. Despite limited information, evaluators must decide whether to consider each job applicant for the next stage in the selection process. When evaluators perceive that Muslims possess unsavory characteristics because of Muslim terrorist activities that evaluators easily retrieve from memory, they may overestimate the probability that Muslim applicants want to harm others.

Integrating the lack of fit model (Heilman, 1983) and the perspectives cited above, evaluators may associate Muslims with violence because of easily recalled acts of aggression Muslims have committed against innocent civilians. As a result of the association, they believe the qualities Muslim applicants possess are incongruent with public safety jobs and view Muslims as unlikeable which in and of itself can result in bias (Krings & Olivares, 2007). During the resume screening process, reviewer stereotypes may impact decision-making because resumes are somewhat ambiguous and often contain incomplete information about an applicant. Given the scant information, reviewers are likely to rely on stereotypes during the evaluation process in an effort to arrive at a judgment of an applicant’s characteristics (see Fisk 1993; Jussim et al., 2015). To the degree they view Muslims unfavorably on characteristics related to warmth and trust, and/or when the warmth evaluation reflects perceived mal intent (Fiske, 2015), resume reviewers may rank Muslims lower than non-Muslim job applicants particularly for public safety jobs. Consequently, they are less likely to offer an interview to Muslims as a function of their low rank.

We believe one salient characteristic that influences evaluator decisions is trust-a component of warmth (Strinić et al., 2020). Biased evaluators may not trust Muslims enough to hire them over non-Muslims for positions that serve the public good and perhaps feel that Muslims are a better fit with other jobs. Given the lack of congruence between mistrust of a person and having that person serve in a security position, we expect evaluators would prefer Muslims for non-security positions over security type jobs that afford job incumbents possessing ill will the opportunity to do harm. Thus, we expect different levels of bias to occur across jobs.

**H1.** Applicant evaluation is a function of the interaction between religion and job type such that:

**H1a.** Evaluators will rank Muslim applicants for a security position lower than non-Muslim applicants. There will be no rank difference for the non-security job.

**H1b.** Evaluators will be less likely to invite Muslim applicants to interview for a security position than non-Muslim applicants. There will be no invite decision difference for the non-security job.

**H1c.** Evaluators will be more likely to rank Muslims higher and be more likely to interview them for the non-security position than the security job.

## Islam and Gender

Whereas our hypotheses up to this point compared Muslims with non-Muslims, we use another perspective as a framework to understand the impact of subsets of demographic variables such as gender within religion on evaluators’ behavior. In our study, this involves describing how evaluators integrate Islam and gender stereotypes in the assessment of the suitability of Muslim men vis-a-vis Muslim women for security and non-security type jobs.

The MOSIAC model (E. Hall, A. Hall et al., 2019) describes how evaluators integrate multiple target stereotypes such as religion and gender and how the evaluator will subsequently behave towards the target as a result of that integration. The model specifies three demographic categories: foundational and two ancillary demographic categories (i.e., intersectional and associated). The foundational demographic category is the category that is similar across, for example, two people whom evaluators compare. The intersectional category is the demographic that evaluators use to distinguish among people in the foundational category. In our comparison of a Muslim man and a Muslim female, Islam is the foundational demographic category and gender is the intersectional demographic category.^2^ Evaluators integrate the foundational and ancillary stereotypes. The resulting integrated stereotype is more pronounced when the ancillary stereotypes are congruent with the foundational stereotype. On the other hand, the strength of the integrated stereotypical belief is attenuated when the ancillary and foundational demographic stereotypes are dissimilar. The integrated stereotype, in turn, impacts the degree to which evaluators notice a target’s behavior, their tolerance for the target’s negative behavior and expectations for the target emitting positive behavior. Perhaps another consequence of the integrated stereotype is the expectation of the likelihood of the target emitting counterproductive behavior.

Based on the MOSAIC model, the intersectional stereotype of men as aggressive has the effect of magnifying the foundational Muslim stereotypes of aggressive, violent, and not trustworthy in the resulting evaluator integrated stereotype. Conversely, as women are seen as less aggressive than men (Sidanius & Pratto, 1999) and the Western female demographic stereotype includes being warm and caring (Fiske, 2017) and nice and sociable (Prentice & Carranza, 2002), the MOSAIC model might predict that the stereotype of a women has the effect of attenuating the impact of the aggressive Muslim stereotype in the resulting integrated stereotype of the Muslim female. If this is the case, we would expect that Muslim women would receive more favorable evaluations than Muslim men for some jobs involving public safety.

Evidence suggests that that some people living in the West view Muslim men in a more negative light than Muslim women. Muslim men are involved in more acts of terrorism than Muslim females and some people claim that Westerners view Muslim men as oppressors and subjugators of Muslim women (Altareb, 1997; Kamalipour, 2000; Slade, 1981). Attitudes of Muslims living in some countries that indicate women should not have equal rights and decision-making freedom on certain issues (Pew Research Center, 2013) support the negative perception. Recent research also documents, that in some instances, discrimination is particularly extant for Muslim males from Africa and the Middle East (Di Stasio et al., 2021). Consistent with the above findings is research documenting that male stereotypes fit their nationalities more than female stereotypes and the difference is more pronounced in countries unfavorably viewed (Eagly & Kite, 1987). As some countries Westerners unfavorably view contain a majority of Muslims, we believe that research is consistent with what we expect our findings to show.

Given the application of the MOSAIC model and the views of Muslim men described in the preceding paragraph, we propose that people view Muslim males as more incongruent with security positions than Muslim females. In turn, that leads us to hypothesize that Muslim gender and job type has an interactive effect on selection decisions.

**H2.** Discrimination in selection decision-making for Muslim applicants is a function of the interaction between gender and job type such that:

**H2a.** Evaluators will rank a Muslim male applicant for a security position lower than a Muslim female applicant. There will be no gender-based rank differences for the non-security job.

**H2b.** Evaluators will be less likely to invite a Muslim male applicant to interview for a security position than a Muslim female applicant. There will be no invite decision differences for the non-security job.

## Trust

A fundamental assumption underlying all of our hypotheses is that raters would view Muslim candidates as less trustworthy than non-Muslim applicants. Indeed, evidence suggests that people in some western countries rate Muslims lower in warmth (Durante et al., 2017) of which trust is a component. We would expect that trust perceptions affecting decision-making would be associated with jobs where trust is a particularly salient characteristic for successful job performance. Similarly, we would expect that evaluators would trust Muslim males less than Muslim females given that more Muslim males are involved in acts of terror than Muslim females. We therefore propose the following hypotheses.

**H3.** Evaluators’ trust Muslims less than non-Muslims.

**H4.** There is a religion by job type interactive effect on trust perceptions. The difference between trust perceptions of Muslim and non-Muslim applicants for the non-security job will be smaller than the difference in trust perceptions for the security job.

**H5.** Evaluators will provide lower trust ratings for Muslim applicants for a security position than for a non-security position.

**H6.** Evaluators will perceive Muslim females as more trustworthy than Muslim males.

## Method

### Participants

One hundred and 20 employees from Western Canadian organizations participated in this research. All participants either currently make or have made employment selection decisions as part of their job. Seventy-five (63%) people from the original sample that had agreed to participate in the project provided data. Missing data on one or more of the non-demographic measures reduced the sample size to 74 (62%) for the analysis of the rank dependent variable, and 73 (61%) for the analysis of the interview invitation variable. The sample included 38 females, 34 males, and two non-respondents to the gender demographic item. The average age was 38.7. There were 53 (81.5%) managers, 4 (6.2%) supervisors, 4 (6.2%) executives, and four (6.2%) reporting “Other” of the 65 people who had indicated their job level.

### Procedure

The first author obtained a list of organizations in the community along with their respective contact people from a local university’s human resource manager. She contacted those individuals as well people in that same community from organizations not on the list and asked them whether they would participate in a research project about managerial employment decision-making. She left the materials with the participants and collected their responses approximately 1 week later.

This experiment had two phases. Participants read one of two job descriptions, reviewed seven applicant resumes, and evaluated each candidate based on information contained in their resumes during the first phase. Participants entered the second phase of the project after returning Phase 1 materials. During Phase 2, they completed a demographic questionnaire and other scales that were part of a larger project. The first author randomly assigned participants to one of two job conditions.

#### Job conditions

We had selected two jobs based on information contained in an on-line job database (O*NET, 2004, May). The two jobs were security guard, and shipping, receiving, and traffic clerk. Both jobs are from the same job zone meaning that they require similar amounts of work-related skill, knowledge, experience, education, and training. The minimum education requirements for both jobs were a high school diploma or a General Equivalent Diploma (GED). The two jobs do not require any previous work-related experience and people can develop job related skills via on-the-job training programs. O*NET (2004, May) also contained the information we used to develop the job descriptions and job specifications.

### Experimental Materials

#### Resume development

The first author had developed seven resumes with the aim of making all seven resumes equivalent in terms of qualifications and experiences. While some researchers have employed one resume and manipulated the potential discriminatory characteristic across the two resumes, we used multiple resumes to reduce the probability of a resume **×** religion confound (see Jackson, 1992). We chose to develop seven resumes for several reasons. We wanted to make the experiment reflect more accurately the number of resumes evaluators might review than studies only using two resumes, to be respectful of the participants’ time, and to mask better the purpose of the experiment than had we only crafted two Muslim and two non-Muslim resumes. All resumes indicated that the applicants received their education in Canada to avoid a possible confound with national origin and contained information about membership in either religious, volunteer, and/or charitable organizations. The first author also used one Muslim male name and one Muslim female name to distinguish the Muslim candidates from the non-Muslim applicants.

Two judges reviewed the resumes after the first author had removed religious based identifying information such as the names of religious-based organizations where the candidates either worked or provided volunteer service. Both judges, with one being the second author, earned doctorates in a human resource management related field and both have experience in personnel selection. Both judges agreed there were negligible differences among the resumes in terms of experiences specified and that they were equivalent regarding applicant KSAs. Through consensus, the two judges selected two resumes to assign to the Muslim applicants. Their goal in matching the resumes to the Muslim applicants was to avoid associating the Muslims with possible markedly positive or negative qualities that would have confounded religion and qualifications. Supplementary Appendix A contains the resumes.

#### Job advertisement

The first author developed two job advertisements. One job advertisement announced the opportunity to apply for a security guard position at a water treatment plant. The second announcement advertised the opportunity to apply for a shipping and receiving clerk position. Both advertisements included an overview of the respective job’s tasks and specifications. Both jobs used the same postal address.

#### Job description

Participants had received one of the two job descriptions in their project materials packet. The description included principal duties, required knowledge, skills and abilities, and a statement indicating that the position requires no previous work-related skill, knowledge, or experience. The description also indicated that applicants needed to possess at least a high school diploma or GED certificate.

#### Pre-test

The first author pre-tested all project materials with several graduate students enrolled in a graduate business program at a western Canadian university. The purpose of the pre-test was to determine whether subjects understood the instructions and materials as well as to ascertain the time requirements of the project’s two phases.

### Dependent Variables

#### Rank

Participants ranked all applicants on their overall suitability for the job on a 1 (Most qualified) to 7 (Least qualified) scale. We reverse coded the scores before analyzing the data to aid interpretation. One’s rank does not suggest whether a particular candidate is qualified for the position in an absolute sense. Rather, rank reflects a qualification-based judgment relative to the other six applicants.

#### Interview invitation score

Participants indicated whether they would extend an interview invitation to each applicant using a No (0) Yes (1) scale. Participants could invite any number of applicants to interview. We believe this variable reflects an absolute qualification-based judgment because it is unlikely evaluators would extend an interview invitation to unqualified people.

#### Trust

Participants rated each applicant on the item, “Does the applicant possess the characteristic ‘trustworthiness’?” on a 1 (Does not possess at all.) to 7 (Clearly possesses.) Likert scale.

### Demographic Variables

Participants indicated their age, gender, education, current position level, number of years of experience with employment selection, and type of employing organization.

## Results

### Descriptive Statistics

Table 1 contains descriptive statistics and variable correlations. Age was positively related to experience (*r* = .58, *p* < .001) and position (*r* = .34, *p* = .007). It was negatively related to the non-Muslim interview invitation score (*r* = −.29, *p* = .02). There were no relations of gender, education, and experience with either rank or interview invitation scores. Muslim trust was negatively related to job type (coded “0” for the non-security job and “1” for the security guard job) and rank.

**Table 1.** Descriptive Statistics and Variable Correlations.

Variable	M	SD	1	2	3	4	5	6	7	8	9	10
1. Job Type	-	-										
2. Age	38.37	10.30	−.19									
3. Gender	-	-	.10	−.06								
4. Education	3.23	1.45	−.11	.18	−.06							
5. Experience	9.72	6.72	.04	.58	−.11	.13						
6. Non-Muslim Rank	4.25	.53	.43	−.09	−.01	−.14	−.01					
7. Muslim Rank	3.36	1.31	−.43	.09	.01	.14	.01	−1.0				
8. Non-Muslim Interview Score	.62	.21	.25	−.29	.09	−.24	−.23	.37	−.37			
9. Muslim Interview Score	.44	.42	−.22	−.13	−.02	.05	−.22	−.67	.66	.07		
10.Trust Non-Muslim	5.43	.85	−.18	.17	.02	.10	.12	.00	.00	.12	.08	
11.Trust Muslim	5.06	1.05	−.37	.19	−.06	.01	.08	−.41	.41	−.10	.21	.61

*Note. n* = 62; Listwise deletion. Correlations greater than ± .25 and .30 have a *p* value < .05 and < .01 respectively. The job type/gender correlation is a phi coefficient; all other values involving either job type or gender are point bi-serial correlation coefficients. Job Type (0 = Shipping and receiving clerk; 1 = Security guard). Gender (0 = Female, 1= Male). Interview Score (0 = No; 1 = Yes. Rank 1 = Least qualified; 7 = Most qualified). We omit calculating means and standard deviations for job type and gender because the mean and standard deviation values of those two variables are not meaningful.

### Religion Comparison-Rank and Job Type

Our first set of analyses tested the hypothesis proposing that raters’ applicant ranking is a function of religion and job type. Unlike some discrimination studies where researchers gathered ratings on only one candidate, our participants evaluated and ranked all applicants. This situation requires a different kind of analysis than what researchers typically use in discrimination studies. To assess Hypothesis 1a, we classified each candidate as either Muslim or non-Muslim based on information the resumes contained. We then calculated the average rank the evaluators gave each applicant and then averaged across applicants within each of the two religious groups. In other words, we obtained an average rank for Muslims and a rank for non-Muslims which served as our dependent variable.

**Figure 1.**
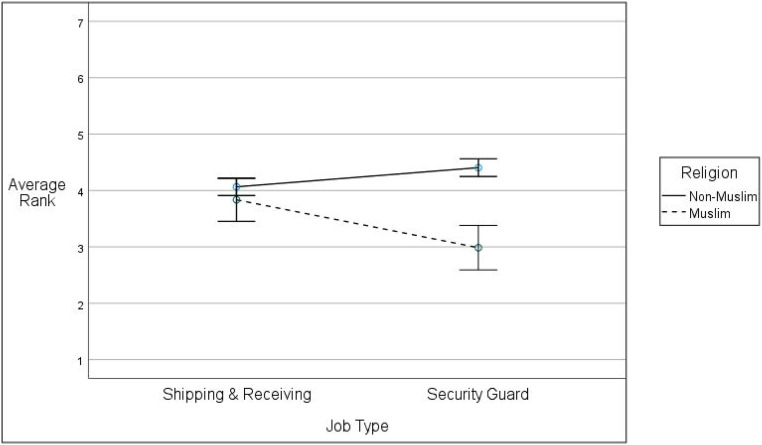
Religion by job type effect on applicant rank.

We conducted a test of within-subjects contrasts to test the first hypothesis by assessing between subject differences based on job type as well as differences in the way each respondent ranked the Muslim and non-Muslim candidates (i.e., whether there were religion-based within-subject ranking differences). Using evaluator average rank of the two religious groups as two different observations, and job type as the independent variable, we interpreted the within-subjects contrasts religion **×** job type interaction term as the degree to which the applicant ranking differed as a function of religion and job type. Table 2 contains the results. Data reveal a religion within-subjects effect that accounted for 20% of the variance in ranking (*F*(1,72) = 17.62, *p* < .001, *partial η*^*2*^ = .197). In support of Hypothesis 1a, the analysis revealed a religion **×** job type interaction term accounting for 12% of the ranking variance (*F*(1,72) = 9.52, *p* = .003, *partial η*^*2*^ = .117). Figure 1 presents the data in graphic form. The graph reveals a negligible difference between the average rank for non-Muslims (*M* = 4.07, *SD* = .50, 95% CI [3.90, 4.23]) and Muslims (*M* = 3.84, *SD* = 1.24, 95% CI [3.43, 4.24]) for the non-security job. There is a marked difference between the non-Muslim rank (*M* = 4.41, *SD* = .45, 95% CI [4.25, 4.56]) and the Muslim rank (*M* = 2.99, *SD* = 1.12, 95% CI [2.61, 3.36]) for the security guard job condition.

**Table 2.** Applicant Rank as a Function of Job Type and Religion.

Source	SS	df	MS	F	Partial eta^2^
Religion	24.20	1	24.20	17.62**	.20
Religion*Job Type	13.07	1	13.07	9.52**	.12
Error	98.89	72	1.37		

*Note. n* = 74; SS are Type 1 sums of squares; ^**^p ≤ .01.

### Religion Comparison-Interview and Job Type

We conducted a hierarchical loglinear analysis (Tabachnick & Fidell, 1989) to assess the pattern of associations among the three dichotomous variables: job type, applicant religion, and interview invitation (Hypothesis 1b). The three-way interaction among the three variables was statistically significant (estimate = −.138, 95% CI [−.236, −.039], Z = −2.745, *p* = .006). Table 3 presents frequency data for the respondents’ interview invitation judgments. The data show the proportion of Muslim applicants invited to interview for the security job (34%) markedly differs from the proportion of non-Muslim applicants invited to interview (65%). However, the differences in proportions were not as pronounced when the interview invitation decision involved the non-security position (Muslims 54%, non-Muslims 58%).

**Table 3.** Job Type × Religion Crosstabulation of Interview Invitation Decision.

	Interview Invitation		
Religion			
Non-Muslim	Yes	No	Total
Job Type			
Shipping and Receiving Clerk	110 (58%)	80 (42%)	190
Security Guard	114 (65%)	61 (35%)	175
Muslim			
Job Type			
Shipping and Receiving Clerk	41 (54%)	35 (46%)	76
Security Guard	24 (34%)	46 (66%)	70

*Note.* Seventy-three participants made decisions on the two Muslim candidates (146 evaluations total) and decisions on the five non-Muslim candidates (365 evaluations total).

### Muslim Evaluation and Job Type

We tested Hypothesis 1c by conducting a one-way ANOVA on the rank dependent variable for the two Muslim applicants to determine whether Muslims were ranked lower for the security job than the non-security job. Results were statistically significant (*F*(1,72) = 9.52, *p* = .003). The average rank the 38 participants assigned to the Muslim applicants for the shipping and receiving clerk job (*M* = 3.84, *SD* = 1.24, 95% CI [3.43, 4.24]) was higher than the average rank the 36 people assigned the Muslim applicants for the security guard position (*M* = 2.99, *SD* = 1.12, 95% CI [2.61, 3.36]).

ANOVA results also reveal differences on the Muslim candidate interview invite decision that support Hypothesis 1c. The average score evaluators assigned to the Muslim applicants for the non-security job (*M* = .54, *SD* = .37, 95% CI [.42, .66] was higher than the average score the evaluators assigned the Muslim applicants for the security guard position (*M* = .34, *SD* = .40, 95% CI [.21, .48]. That difference is statistically significant (*F*(1,71) = 4.73, *p* = .033, *partial η*^*2*^ =.062).

### Muslim Gender Evaluation and Job Type

**Figure 2.**
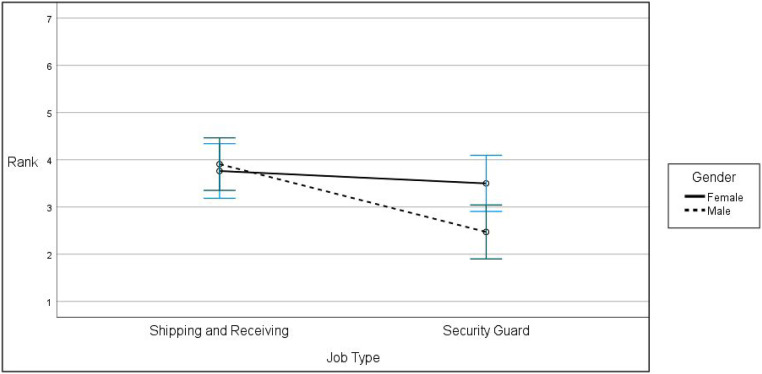
Gender by job type effect on Muslim applicant rank.

Hypothesis 2 specifies a gender **×** job type interaction effect on rank where the level of bias evaluators exhibited toward Muslim men relative to Muslim women differs as a function of job type. We conducted a test of within-subjects contrasts to evaluate the hypothesis with Muslim applicant gender being the within-subjects factor. Table 4 contains the results and Figure 2 depicts the data in graphic form. The data reveal a gender **×** job type interaction term accounting for 5% of the variance in rank (*F*(1,72) = 3.79, *p* = .055, *partial η*^*2*^ = .05). Figure 2 illustrates the relation. The Muslim male’s rank (*M* = 2.47, *SD* = 1.58, 95% CI [1.94, 3.01] was modestly lower than the Muslim female’s rank (*M* = 3.50, *SD* = 1.61, 95% CI [2.96, 4.04] for the security guard job. There was little difference between the Muslim male rank (*M* = 3.91, *SD* = 1.84, 95% CI [3.30, 4.51] and the Muslim female rank (*M* = 3.76, *SD* = 1.94, 95% CI [3.12, 4.40] for the non-security position.

**Table 4.** Muslim Applicant Rank as a Function of Job Type and Gender.

Source	SS	df	MS	F	Partial eta^2^
Gender	6.70	1	6.70	2.00	.03
Gender*Job Type	12.71	1	12.71	3.79	.05
Error	241.46	72	3.36		

*Note. n* = 74, SS are Type I sums of squares.

We also ran a Friedman test for each job condition. Setting alpha at .025 to control for Type 1 error, we found no difference in the rank participants assigned to the Muslim male and the Muslim female in the non-security job condition (*χ*^2^(1) = .03, *p* = .869). We found a marked difference in the rank participants assigned to the two applicants for the security guard job condition (*χ*^*2*^(1) = 7.11, *p* = .008). These data support H2a.

Hypothesis 2b posited that evaluators will be less likely to invite a Muslim male applicant to interview for a security job than a Muslim female applicant but there would be no difference for the non-security job. We conducted a hierarchical log-linear analysis to test the association among gender, job type and interview decision for the two Muslim applicants. The interaction effect of job type, Muslim gender, and interview decision was not statistically significant (estimate = .075, 95% CI [−.091, .240], Z = .883, *p* = .377). Table 5 contains crosstabulation data. These data show little differences in the proportion of interview invitations for the Muslim female applicant compared to the Muslim male applicant. In sum, we did not find support for Hypothesis 2b.

**Table 5.** Muslim Applicant Job Type **×** Gender Crosstabulation of Interview Invitation Decision.

	Interview Invitation		
Gender			
Male	Yes	No	Total
Job Type			
Shipping and Receiving Clerk	21 (55%)	17 (45%)	38
Security Guard	10 (29%)	25 (71%)	35
Female			
Job Type			
Shipping and Receiving Clerk	20 (53%)	18 (47%)	38
Security Guard	14 (40%)	21 (60%)	35

*Note.* Thirty-eight participants made decisions on the two Muslim candidates for the shipping and receiving clerk position (76 evaluations total). Thirty-five participants made decisions on the two Muslim candidates for the security guard position (70 evaluations total).

### Religion Trust Ratings Differences

We conducted a test of within-subjects contrasts to determine whether the evaluators rated Muslim applicants lower on trust than non-Muslim applicants (Hypothesis 3) and whether job type affected that relation (Hypothesis 4). We observed a religion within-subjects effect that was statistically significant (*F*(1,73) = 9.35, *p* = .003), partial η^2^ =.113). The Muslim applicants received lower trust ratings (*M* = 5.09, *SD* = 1.03, 95% CI [4.85, 5.32] than non-Muslims (*M* = 5.40, *SD* = .85, 95% CI [5.20, 5.59]). The trust by job type interaction effect was not statistically significant (*F*(1,73) = 2.66, *p* = .107, partial η^2^ =.035). Although, evaluators rated Muslim applicants lower than non-Muslims on trust, the impact of the type of job the applicants applied for did not affect the evaluators’ trust perceptions. These data support Hypothesis 3 but do not support Hypothesis 4.

### Muslim Trust by Job Type

Hypothesis 5 specified that Muslim applicants for the security-related position would receive a lower trust rating than they would for the non-security position. Results from a regression analysis reveal that the mean trust score of Muslim applicants for the security position (*M* = 4.81, *SD* = 1.02, 95% CI [4.46, 5.15] was lower than the mean trust rating of Muslim applicants for the non-security position (*M* = 5.35, *SD* = .98, 95% CI [5.03, 5.66]). That difference was statistically significant (*F*(1,73) = 5.46, *p* = .022, *R*^*2*^ = .07) and supports Hypothesis 5.

### Muslim Gender Trust Comparison

We hypothesized that raters would perceive the Muslim female as more trustworthy than the Muslim male. The mean difference between the female trust rating across the two jobs (*M* = 5.05, *SD* = 1.11, 95% CI [4.80, 5.31] and the male trust rating (*M* = 5.12, *SD* = 1.21, 95% CI [4.84, 5.40] was not statistically significant based on the results of the test of within-subjects contrasts (*F*(1,74) = .29, *p* = .59, *partial η*^*2*^ = .01). We also found no Muslim gender by job type effect and therefore cannot support Hypothesis 6.

## Discussion

Our research suggests that religion influences employers’ employment decisions for some jobs. Unlike the non-security job, Muslims were ranked lower and less likely to be invited to interview for a security position than non-Muslims. Given the salience of events involving Muslims and terrorism, decision-makers may believe hiring a Muslim for a job involving public safety is riskier than hiring a non-Muslim. This is consistent with our data showing that evaluators trusted Muslims less than non-Muslims and that Muslim applicants for the security job received lower trust ratings than Muslim applicants for the non-security job.

We obtained mixed support for the proposed interactive effects of gender and job type for the Muslim applicants. While we observed a modest interactive effect for rank, the lack of an interactive effect on the interview invite decision may be the result of a floor effect. Both Muslim applicants were among the lowest ranked applicants for the security guard position and therefore were less likely to receive an interview invitation than non-Muslims. Perhaps we were unable to detect gender differences in an interview invitation between the two Muslim applicants given their low rank. The finding highlights that, at least for a security position, religion appears to have had a larger effect on the interview invite decision than gender. When the same level of salience does not exist for a characteristic such as gender as it does for religion, religion should more heavily influence decision-making.

Our research findings are consistent with others’ research. Derous et al. (2011) examined job type effect on biased judgments. One of their findings was that being an Arab female had a more negative impact on resume evaluations than combinations of ethnicity and gender for high status jobs. Although we observed a slight disadvantage to Muslim males compared to Muslim females, our findings and those of Derous et al. (2011) highlight the importance of understanding what decision-makers see as salient characteristics associated with job performance to understand better how those decision-makers react to applicant demographic composition. Our results also support models specifying how intersections of applicant characteristics such as race and immigrant status can affect hiring decisions and other judgements (Akinlade et al., 2020).

Our findings are important because it supports the notion that when evaluators lack detailed information about a candidate, as is typical when reviewing a resume, they may form judgments about the candidate based on that candidate’s minority status. This in turn, increases the likelihood that evaluators will determine that a minority candidate is congruent for some jobs but not others. A disproportionally small number of minorities associated with certain jobs reduces the visibility of those minorities in those jobs. Lacking visibility in some occupations may strengthen the stereotype that members of particular minority groups aren’t qualified for those occupations. The lack of visibility leads to a vicious cycle that perpetuates the stereotype that members of certain groups are incongruent with certain occupations (Eagly & Koenig, 2021). Societal underutilization of skills is the result of this vicious circle.

Our research points to the importance of engagement or lack thereof on judgement formation and behavior. When there is little personal experience with another culture due to a lack of direct contact, people are more likely to form stereotypes (Fiske, 1993). It is through mutual acceptance and desire for both minority and majority cultures to interact with each other that enable different cultures to integrate (Berry, 2021). The personal experiences that develop from this integration increases the information known about the other culture thereby attenuating the influence of stereotypes on judgments and behavior. Indeed, Bessudnov and Shcherbak (2020) speculated that increased contact was a plausible reason for their finding of less discrimination in multi-ethnic locals than in more homogeneous environments or in settings where ethnicities were separated from each other.

It would be erroneous to take our findings and conclude that the results are incongruent with gender and job type research because comparing our findings with that literature is comparing apples to oranges. Perhaps gender, which is typically salient in the evaluation of female candidates for a male-type job such as a security guard, does not have the impact it normally would if evaluators are assessing candidates of a particular religion. Rather than contradicting previous gender discrimination research, we feel our work extends that literature by attempting to explain how evaluators might assess competing incongruent person-job fit perceptions. Perhaps trust or lack thereof is associated more with people of certain religions than gender.

### Contributions

One contribution of our work is the support and extension of the MOSAIC model. The model specifies that the integration of functional, intersectional demographic, and associated demographics leads to the formation of templates that impact the evaluators noticing the target’s behavior, the tolerance level for negative behavior, and the expectation of positive behavior. While the tolerance for negative behavior might imply that there is some expectation that counterproductive behavior will occur as a function of stereotype integration, perhaps it is useful to specify in the model high/low expectations of negative behavior to the same degree as the specification of high/low positive expectations. When an evaluator believes that there is a high expectation for a job applicant emitting negative behavior that can cause harm, that individual won’t hire the applicant thereby eliminating the possibility of observing and tolerating counterproductive behavior. Thus, we expand MOSAIC’s criterion domain to include high/low expectations of negative behavior in addition to the model’s expectation of positive behavior because it is possible for individuals to engage in both positive and undesirable behaviors.

We also contribute to the intersectionality research. We show that it is necessary to understand the specific context under which people apply their perceptions to understand better the degree to which combinations of demographics affect perceiver behavior. It is insufficient to merely examine how combinations of characteristics affect selection because the demographic differences we observed were a function of the job. Thus, not only does the researcher need to understand stereotypes associated with demographic combinations, what also needs to be understood is how the demographic combinations relate to the job under consideration in terms its requirements and/or the environment where the job occurs.

We extend the religious discrimination literature. Most of the early literature on alleged Muslim discrimination did not provide much, if any, evidence to support claims of mistreatment. Recent research documents discrimination and we extend the data-based religious discrimination studies by demonstrating where discrimination is more likely to occur and offer a possible explanation as to why it occurs. Discrimination may not occur for all jobs. Rather it appears that discrimination is more likely to occur when stereotypes appear to be incongruent with salient job requirements than for jobs that do not possess that salient job requirement. Another notable feature of our research is the use of experimental procedures that allows for inferences of causality. We also used participants who make selection decisions as part of their jobs thereby increasing the results’ external validity than had we used students or people with no selection experience.

### Limitations and Future Research

There are boundary conditions that limit the generalizability of the results. Perhaps the participants responded the way they did because of ethnicity and/or immigration status instead of religion despite specifying in our experimental materials the candidates resided and educated in Canada. Indeed, reviews and research documents discrimination based on those two alternative explanations (Agerström, et al., 2012; Derous, et al., 2011; Esses, et al., 2014) and is consistent with perceptions that Middle Easterners possess relatively lower warmth compared to other groups (Lee and Fiske, 2016).

The design we used likely constrained interpretation of the test comparing the two Muslim applicants because those ranks were not independent. Both rankings were affected by how participants ranked the non-Muslim applicants. Given that participants ranked the non-Muslims higher than the Muslims for the security guard position, the range of possible differences in the ranking between the two Muslim applicants was attenuated. Even if we excluded non-Muslims, there are alternative ways of comparing the two Muslims. One example is to have participants rate the applicants on a variety of job-related qualifications as well as the likelihood to emit counterproductive behavior.

While some people claim that few North Americans distinguish between Arab, Muslim, Persians, and other residents of the Middle East (Kamalipour, 2000; Kenny, 1975), other researchers found that religious affiliation explains discriminatory outcomes based on ethnicity (Cheung, 2014; Heath & Martin, 2013). Given what appear to be conflicting beliefs, researchers need to conduct additional investigations to assess the unique and interactive contributions of ethnicity and religion on employment discrimination.

We examined only one job involving public safety. There are many other public safety-type jobs such as police officers, firefighters, officials screening passengers before boarding airplanes, and airport baggage inspectors. Job suitability perceptions may differ among those occupations and future research should investigate possible perceptual differences to assess the generalizability of our findings.

One alternative explanation for the results is that there were marked differences in the applicants’ qualifications that the two HRM experts did not detect and that the two weakest resumes were the ones that by chance the researchers assigned to the two Muslim candidates. While the odds of that occurring is 16.67% and the explanation does not explicate the lack of differences we observed in decisions among Muslim and non-Muslim applicants for the non-security position, this study needs to be replicated using different experimental materials.

Our ratio of Muslims to non-Muslims was not equivalent. Heilman (1980) reported that the majority-minority applicant ratios in the applicant pool can affect employment decisions with the greatest effect occurring when the minority applicants represent 25% or less of the applicant pool. While our ratios approached that percentage in the religion-based hypotheses we evaluated (i.e., 29%), that explanation doesn’t explain the lack of differences we found for the non-security position. Future research should examine religious difference effects with similar ratios.

### Practical Implications

Mangers and researchers have focused primarily on race and gender in employment discrimination research. Our findings indicate that practitioners need to be aware that religious employment discrimination exists as well as highlighting the need to include religion in discrimination reducing interventions. Trainers could have managers examine their own beliefs about religious people and how those biases can impact applicant evaluations for certain jobs. Indicating how news events can affect their perceptions should be included in the program. It is also incumbent upon trainers to have trainees realize that discrimination is not an all or none response. It might be present in applicant evaluations for some jobs but not others. Thus, trainees need to understand that they may make fair and bias free judgments in some situations but not in others. Trainers also need to raise trainees’ awareness that religious biases may override the biases they hold on other characteristics such as gender. Perhaps incorporation of these features in training interventions will reduce the occurrence religious discrimination. In turn, that would decrease the social and economic costs to both applicants and organizations that accompany litigation stemming from both unfair treatment and costs associated with hiring less qualified people.

## Supplemental Material

Supplemental Material - Job Type, Religion, and Muslim Gender as Predictors of Discrimination in Employment Settings Supplemental Material for Job Type, Religion, and Muslim Gender as Predictors of Discrimination in Employment Settings by Kazhal Mansouri and Richard Perlow in Psychological Reports

## Notes

1 Discrimination as used in this paper refers to unfair discrimination as opposed to fair discrimination. Fair discrimination occurs when decision-makers discriminate among applicants in terms of expected job performance derived from valid selection instrument scores assessing job-related criteria.

2 The associated demographic category is connected with another demographic category. Hall et al. (2019) illustrate the concept with race and gender. Black females are associated with the male demographic category whereas white women are not. Thus, whereas white women’s stereotype is associated only with those of women, the stereotype of a black women is derived from an integration of the female and male stereotypes.
