# Supplementary material for: Job Type, Religion, and Muslim Gender as Predictors of Discrimination in Employment Settings
Source: Psychol Rep. 2022 Oct 12;127(3):1355–80. doi: 10.1177/00332941221092666 (PMC11067403; doi:10.1177/00332941221092666)
Supplement: Supplemental Material - Job Type, Religion, and Muslim Gender as Predictors of Discrimination in Employment Settings [file sj-pdf-1-prx-10.1177_00332941221092666.pdf]

Appendix A

Resumes

ROBERT MCKINNEY

Address: 34 Dallas Av., Kitchener, ON.

Tel: (519) 346-1582

EXPERIENCE

OFFICE CLERK

Astro Insurance Company (May 2003 - Present)

Answered telephones; directed calls and took messages; operated office machines such as photocopiers and scanners, facsimile machines, and voice mail systems; answered customers' questions.

PARKING ATTENDANT

Kitchener General Hospital (Aug. 2001- Mar. 2003)

Greeted customers; calculated parking charges; collected fees; directed motorists to parking areas or parking spaces; issued ticket stubs; positioned and removed barricades to open or close parking areas; parked and retrieved customers' automobiles.

TICKET TAKER

Cinema Silvercity Kitchener (May 2001- Aug. 2001)

Greeted patrons. Examined tickets or passes to verify authenticity. Refused admittance to undesirable persons or persons without tickets or passes.

VOLUNTARY WORK

Voluntary work for United Church of Canada (June 2000 - Jan. 2002)

I coordinated junior high school aged youth group programs at the church.

EDUCATION

Graduate from Kitchener High School, Kitchener, ON. (Sept. 1999 – June 2003)

GPA: 2.93

INTERESTS

Swimming, playing hockey, camping

FATEMEH HAJI

Address: 11 Knightswood St., Winnipeg, MB.

Tel: (204) 346-1582

EXPERIENCE

CUSTOMER SERVICE REPRESENTATIVE

Dairy Queen (Jan. 2003 - Mar. 2004)

Greeted customers and took orders; helped customers to select menu items; placed orders in the kitchen; prepared itemized bills and accepted payments. *

DIETARY AID

Victoria General Hospital (May 2001 - Dec. 2002)

Assembled patient food trays; placed food servings on plates and trays according to instructions; examined trays to ensure that they contain required items; loaded trays with accessories such as napkins; monitored food distribution; ensured that meals are delivered to the correct patients and that guidelines such as those for special diets are followed. I reported directly to the chef.

SERVER

Earls Restaurant (Apr. 1999 - Mar. 2001)

Explained how various menu items were prepared. Informed customers of daily specials. Prepared checks that itemize and total meal costs and sales taxes. Presented menus to patrons and answered questions about menu items and made recommendations upon request. Removed dishes and glasses from tables or counters.

VOLUNTARY WORK

Voluntary work for Muslim Women Association, Winnipeg, MB. (Nov.1998 - Present)

Participate in every Friday praying at the local Mosque; prepare food for attendants; collect nonperishable foods and distribute to low-income Muslim families.

EDUCATION

Graduated from Vincent Massey High School, Winnipeg, MB. (Sept. 2000 - June 2004)

GPA= 3.18

INTERESTS

Reading, swimming, jogging

MOHAMMAD HANANI

Address: 177 Truesdale Dr. E., Regina, SK.

Tel: (306) 332-5743

EXPERIENCE

CUSTOMER SERVICE & REPRESENTATIIVE

Wal-Mart (Feb. 2002 - Jan 2004) Greeted customers and ascertained what customers wanted; opened and closed cash registers; performed tasks such as counting money, separating charge slips, and coupons, balancing cash drawers, and making deposits; helped locate or obtain merchandise based on customer needs and desires.

COOK

Burger King (June 1998 - Nov. 2001)

Cleaned food preparation areas. Cooked and packaged batches of food which are prepared to order or kept warm until sold. Maintained sanitation, health, and safety standards in work areas.

FOOD PREPARATION

Boston Pizza (Jan. 1998 - Jul. 1998)

Assisted cooks and kitchen staffs. Carried food supplies, equipment, and utensils to and from storage and work areas. Cleaned work areas, and equipment. Cut, sliced and/or grinded meat, poultry, and seafood to prepare for cooking. Distributed food to waiters and waitresses.

VOLUNTARY WORK

Active member of Regina Muslim community (Sept. 2000 - Present)

Plan gatherings on Muslim Holy days and do spiritual speeches at gatherings. Help new Muslim immigrants to settle in the area. Show them how they can obtain Muslim meats and foods so they can adhere to their beliefs.

EDUCATION

Passed General Equivalency Diploma in Nov. 2003.

Attended Regina High School, Regina, SK. (Sept. 1999 - June 2003)

INTERESTS

Gardening, basketball, skating

JOSEPH LE CLAIRE

Address: 48 rue Antonin-Campeau, Montréal, QC.

Tel: (450) 688-3091

EXPERIENCE

GAS STATION ATTENDANT

Gas King (May 2002 – May 2004)

Served customers; computed and recorded totals of transactions; accepted payments; pumped gas; checked tire pressure; cleaned, lubricated, and adjusted compressors; watched gauges, dials, or other indicators to make sure machines were working properly; closed store at the end of my shift.

CASHIER

Zellers (July 2001 – May 2002)

Used cash register to record sales; verified identity checks, and processed credit cards; attached price tags to goods; answered phones, and answered customers' questions; provided information on procedures or policies.

LOBBY ATTENDANT

Cinéma du Parc (Jan. 2001 – July 2001)

Assisted patrons in finding seats. Directed patrons to restrooms, concession stands and telephones. Provided assistance with patrons' special needs, such as helping those with wheelchairs.

VOLUNTARY WORK

Volunteer work for World Vision (Sept. 2000 – Feb. 2003)

Collected information of young children from poor countries; filed their pictures and information; answered phone calls with regard to child sponsorship.

EDUCATION

Graduate from Montreal High School, Montreal, QC. (Sept. 1998 – June 2002)

GPA= 2.98

INTERESTS

Bowling, biking, watching TV.

ANN HINTON

Address: 415 Erin Grove SE, Calgary, AB.

Tel: (403) 669-5130

EXPERIENCE

OFFICE CLERK

University of Calgary Admissions Office (June 2001 – April 2004)

Filed documents; helped with registrations; typed letters and documents; answered phones; operated office machines; recorded data and other information in electronic database

COOK AND CASHIER

Arby's Fast Food Restaurant (Oct. 2000 - Mar. 2001)

Greeted customers; took customer's orders, prepared sandwiches, and served meals. Operated large-volume cooking equipment such as grills, deep-fat fryers, or griddles. Prepared and served beverages. Processed cash and credit /debit card payments. Operated cash registry.

CAFTERIA WORKER

University of Calgary Cafeteria (Sep. 1999 – May 2001)

Took orders. Prepared meals and beverages and served to the customers. Cleaned the kitchen and cafeteria.

VOLUNTARY WORK

Voluntary work for St. Michael Church (July 1997 - Present)

Conducted office/clerical services for the church.

EDUCATION

Graduated from Central Memorial High School, Calgary, AB. (Sept. 1999 – June 2003)

GPA = 3.1

INTERESTS

Shopping, watching movies, painting.

JENNIFER ANDREWS

Address: 1852, 89 Ave. NW, Allensville, ON.

Tel: (705) 465-1748

EXPERIENCE

LAUNDERY WORKER

Allenville Laundry (Feb. 2003 – Mar. 2004)

Received and marked articles for laundry or dry cleaning with identifying code number or name. Loaded articles into washer or dry cleaning machine. Started washer, dry cleaner, drier, or extractor. Removed articles from dryer; folded wrapped or hung items for airing out, pick up or delivery. Sorted and counted articles.

CASHIER

Pet Cetera (June 2001 – Feb. 2003)

Answered customers' questions; took customer orders; entered the orders in cash register; p[laced items in a bag; maintained clean and orderly checkout areas; counted money; made change; accepted credit/debit card; reconciled cash and receipts at the end of the night.

AMUSMENT & RECREATION ATTENDANT

Adventure on Wonderland, London, ON. (Mar. 2000 - Aug. 2000)

Provided information about facilities, entertainment options, and rules and regulations. Rented, sold, or issued sporting equipment and supplies such as bowling shoes, and golf balls. Operated cash register and conducted cash/debit transactions. Directed patrons to rides, seats, or attractions. Fastened safety devices for patrons, or provided them with directions for fastening devices.

VOLUNTARY WORK

Voluntary work for Red Cross (Oct. 1999 – Aug. 2003)

I was in Elderly Escort Program. I accompanied local residents to their doctors' appointments and brought them back. I also helped in fund raising programs.

EDUCATION

Sullivan High School graduate, Allensville, ON. (Sept. 2000 – June 2004)

GPA: 3.0

INTERESTS

Playing Soccer, camping, hanging out with family and friends

PAT ROY

Address: 1676 Frances St, Vancouver, BC.

Tel: (604) 325-1018

EXPERIENCE

CUSTOMER SERVICE

Video Head Quarters (Feb. 2001 - Aug. 2003)

Kept records of transactions; prepared rental forms; obtained customer signature and other information such as required licenses; received, examined, and tagged articles to be altered; cleaned, stored, repaired, inspected and adjusted rental items to meet needs of customers; explained rental fees, policies and procedures; operated cash register.

OFFICE CLERK

Vancouver Community College Administration office (Sept. 1999 - Dec. 2000)

Compiled, copied, sorted, and filed records of office activities, business transactions, and other activities; computed, and recorded data and other information, such as records or reports; opened, sorted and routed incoming mail; answered correspondence, and prepared outgoing mail.

COUNTER ATTENDANT

Garibaldi Park Concession Store (Mar. 1999 - Sept. 1999)

Took customers' orders and wrote ordered items on tickets. Gave ticket stubs to customers to identify filled orders. Prepared food using standard formulas. Served food, beverages, or desserts. Wrapped menu items. Collected cash from customers. Operated cash register.

VOLUNTARY WORK

Voluntary work for St. Paul's Church (Apr. 1997 - Dec.1998)

Babysitting children while their parents were attending services.

EDUCATION

Passed G.E.D. test (July 2003)

Attended West Vancouver High School, Vancouver, BC. (Sept.1999 - June 2001)

INTERESTS

Golfing, fishing, swimming
